# Halloysite in Different Ceramic Products: A Review

**DOI:** 10.3390/ma14195501

**Published:** 2021-09-23

**Authors:** Paraskevi Lampropoulou, Dimitrios Papoulis

**Affiliations:** Department of Geology, University of Patras, 26504 Rio, Greece; papoulis@upatras.gr

**Keywords:** halloysite, halloysite ceramics, SiAlONS, HNTS

## Abstract

The increased demands of our rapidly developing way of life lead to the broadening of the ceramic market among other effects. Due to the advanced ceramic properties of halloysite and its abundance, combined with its good synergistic effect with other materials, it has been investigated for multifarious possible applications to produce traditional and advanced ceramics as well as ceramic composites. In this review, a substantial number of studies by several investigators into halloysite-based ceramics were are summarized. The possibilities and limitations of different halloysite-based ceramic materials for future applications are also discussed in this manuscript and new fields of research are proposed. The summarization of published results indicates a constant scientific interest in halloysite-based traditional ceramics and new potential uses in the future. Additionally, investigations on different novel ceramic composites with low cost halloysite nanotubes (HNTS) have rapidly increased, covering different scientific and technological areas. On the other hand, research into advanced ceramics (SiAlONS) has been pursued due to its highly cost effective technology treatments on a large scale.

## 1. Introduction

Nowadays, the broad ceramic market exceeds a value of USD 250 billion while growing at an annual rate of ~8.5%, due to the increased demands of our rapidly developing way of life and technology. In fact, ceramics cover a tremendous field of applications and products (e.g., construction and building materials, advanced materials, refractories, earthenware, etc.) attending to our everyday life and development [1,2,3,4,5]. As it is that known clay minerals possess a significant share of the ceramic industries in the production of several materials (whitewares, glass ceramics, refractories, monolithic, ceramic membranes, SiAlONS, etc.), Refs. [1,6,7,8,9].

**Figure 1:**
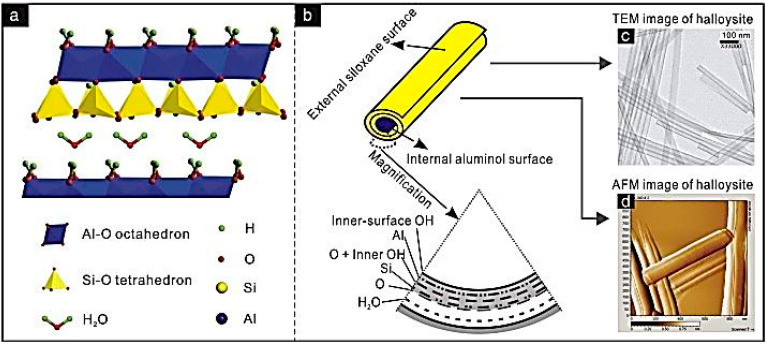
Schematic diagram of (**a**) the crystalline structure of Hal-(10 Å), (**b**) the structure of Hal particle, (**c**–**d**) TEM and AFM images of Hal. [10].

**Figure 2:**
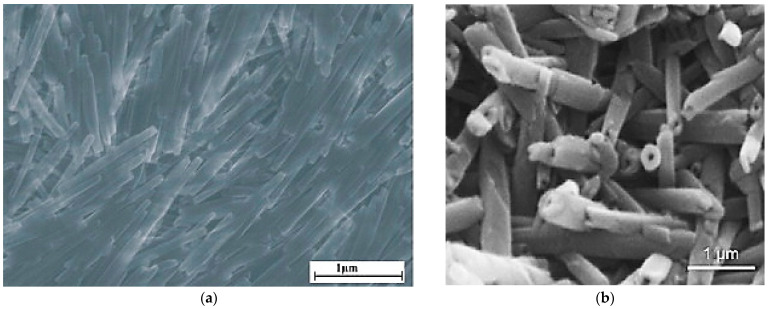
(**a**,**b**) Scanning electron microscope (SEM) images of tubular halloysite mineral on a thin surface section [11] and on a fractured one [12], respectively.

Halloysite is a clay mineral of the kaolinite group that is found in two forms, the hydrated form (Halloysite—10 Å) and the dehydrated one (Halloysite—7 Å) (Figure 1) [10]. It is formed in different several environmental conditions after weathering or hydrothermal alteration of different geological lithotypes. The dominant tubular morphology of halloysite (Figure 2) is derived mainly from altered feldspars or biotite minerals, while alteration of the amorphous phase in volcanic rocks leads to the halloysite occurrence in spheroulite forms [11,12,13].

Halloysite is a common clay mineral and therefore halloysite deposits have been discovered all over the world (China, New Zealand, Brazil, USA, France, Algeria, Turkey, etc.) [14,15,16]. Commonly, in these deposits, halloysite coexists with other clay minerals, quartz, feldspars and oxides in different proportions. Halloysite clay, mainly due to the high Al_2_O_3_ content (>30 wt%), plasticity, the whiteness and non-toxicity, has been widely used globally by ceramic industries for the production of traditional and advanced ceramics, while Asia is considered the main halloysite-based ceramic industrial market [17,18]. Moreover, it is extensively studied for possible applications in different industries for products like polymers, cosmetics, medical products, agriculture materials, nanofillers, catalytic support materials, nanocontainers, adsorbents and immobilizers of toxic metals, anticorrosion agents, etc. [15,19,20,21,22,23,24,25].

The present work constitutes an effort to sum and review previous publications concerning halloysite-based traditional and advanced ceramics. Moreover, this study includes some of the most recent publications dealing with the use of halloysite in new more complex syntheses both with ceramic and no ceramic characteristics (e.g., metallized-ceramic materials or polymer–ceramic materials), used for special industrial applications. For this reason, they have been also included in this review research, named as “ceramic composites”.

## 2. Methodology

The selection of published researchers for this review drew on databases of updated abstract and citation data of peer-reviewed literature and convenient search tools. In order to cover the current scientific topic, the keywords “halloysite” and “ceramics” were used in the bibliographic search. After collection of related published literature, most of them were classified according to the halloysite raw materials’ (potential) use in traditional ceramics, advanced ceramics or in other ceramic composites. In each category the results are presented in chronological order up to the most recent available.

## 3. Halloysite

The world supply of Halloysite clays is in excess of thousand tons per year. Due to their availability and, the fact that are usually found in surficial deposits, they are characterized as low-cost raw materials for ceramics. Halloysite clay price mainly depends on its mineralogical composition. As a result, high amounts of halloysite nanotubes are radically increasing its applications and therefore its price. It should be noted that halloysite nanotubes are significantly cheaper compared with other nanotubes (e.g., carbon nanotubes) and nowadays they are strongly preferred in many advanced ceramic composites [26,27].

The term meta-halloysite is an old term that should not be used anymore and should be replaced by the term halloysite 7 Å. Despite that, in some recent papers the term meta-halloysite is still used. Both halloysite mineral forms (halloysite 7 Å and halloysite 10 Å) have monoclinic crystal symmetries. The dominant tubular morphology of halloysite (while it can be found in other morphologies) is the main difference between halloysite and kaolinite. It should be noted that halloysite is the only clay mineral that has a tubular morphology (nanotubes) as its usual form.

## 4. Summarizing Papers and Discussion

The most frequently identified information from this review and future perspectives are discussed below:

### 4.1. Traditional Ceramics

Traditional ceramics were derived mainly from natural raw aluminosilicates including several products, e.g., tableware, clay bricks, tiles, refractories, earthenware, etc. Τhis section includes previous investigations on the characterization of natural halloysite containing clays for their potential use in traditional ceramics as well as published studies on ceramic products from halloysite-containing clays used mostly for common traditional uses (Table 1).

Gualtieri and Bertolani [28] produced ceramics from halloysite-rich clays (Central Italy) after slow and rapid firing at 1200 °C and 1085–1195 °C, respectively. Raw materials were comprised mainly of halloysite 10 Å, siliceous amorphous phase and feldspars. Generally raw materials transformed to mullite and cristobalite. The metaphase of mullite-II was formed at 1000–1175 °C and at 1025–1200 °C during the slow and rapid sintering, respectively. According to the authors the initial disordered structure of halloysite increased the rate of mullite-II development. Moreover, advanced mullitization was detected in samples with higher amounts of halloysite (>57 wt%) in raw materials while the amorphous siliceous phase of bulk clay material contributed to the cristobalite formation. The last was not or was scarcely detected up to 1085 °C during the rapid sintering. Shelest, a, b [29,30] investigated the potential use of different clays (Russian area) in the refractory production. He concluded that in case of the kaolin mixture, containing halloysite up to 40%, the presence of kaolinite and montmorillonite minerals contributed positively to the sintering processing, while he proposed that additions should be used in order to be produced to succeed chamote and high-Alumina refractories in steel industries. Sumi et al. [31] investigated new synthesized cordierite ceramic products from kaolinite or halloysite clay and magnesium hydroxide after firing at 1350 °C in a reducing atmosphere. The results show that mixtures of tabular kaolinite and synthesized tabular magnesium hydroxide produced dense cordierite products after sintering whilst lower quality fired samples from needlelike halloysite and synthesized tabular magnesium hydroxide were produced, due to their different crystal morphology and consequent lower reacted specific surface areas and insufficient sintering.

After a time gap of twelve years, Elueze et al. [32] studied the physicochemical properties and mineralogical compositions of kaolinite–halloysite clay materials derived from the Yaounde area, southern Cameroon and concluded that according to the industrial technical standards they could be used to the production of burnt bricks, ceramic and earthenware products. Later, Imai et al. [33] produced nano-mullite ceramics after spark plasma sintering of halloysite-rich clay powder with low impurities at 1300 °C. The final products were characterized by a dense texture with precipitated tubular nano-mullite of 20–30 nm crystal size exhibiting 70–80% translucence at 100–2500 nm wavelength, whilst the effect of different halloysite types (7 and 10 Å), their morphology and particle size did not correlate with mullite crystallization. Different prepared starting mixtures of clays with and without alumina were investigated by Tezuka et al. [34] according to their thermal behavior and final mineral compositions, after sintering up to 1500 °C. The results revealed that both final products derived from kaolinite-rich clay and halloysite-rich clay contained the same amounts of the new mullite phase, approximately 65 wt% which first appeared after 1100 °C in their matrix. On the other hand, in ceramic from a fired kaolinite–alumina mixture, higher amounts of mullite (65 wt%) were detected compared to that from raw materials’ halloysite–alumina (56 wt%). This is due to the increased content of low melting impurities and reduced cristobalite of starting kaolinite clay compared to those in the halloysite clay, leading to the enhanced active thermal reactions with alumina.

Septawendar et al. [35] investigated halloysite-rich clay raw materials derived from Tanjung Morawa of Indonesia. They indicated that the raw materials consisted of halloysite, feldspar, montmorillonite, mica and quartz, transformed to mullite, cristobalite and a-quartz after firing up to 1000 °C. The rate of bending strength of the ceramic body was approved equal to the mullite+cristobalite increase. Consequently, in 2008 Pialy et al. [36] worked in the Lembo field area of Cameroon and published their research on characterization of kaolinite–halloysite clays. They revealed a variety in terms of their compositions, crystallinity and consequent potential uses in a broad field of commercial ceramics but not for applications based οn purity. Halloysite dehydration in samples occurred between 150 and 400 °C. Iron participation both in halloysite and kaolinite crystals possibly enhanced their disordering during the sintering processing and reaction kinetics, indicating mullite nucleation at 988 °C. The same year, Dondi et al. [37] investigated kaolins derived from different Patagonia deposits. These materials exhibited a variety in terms of mineralogical compositions, microstructures and physico–mechanical properties influencing the technological demands on sanitaryware and porcelain stoneware production. They indicated that plasticity was increased with the kaolinite group minerals (kaolinite, halloysite) and expanded mineral increase as well as the particle size decrease, while kaolinite+halloysite amounts (max 63 wt%) enhanced strongly the ceramic slip casting. Moreover, tubular crystals of halloysite affected the slip rheological properties of clays, increasing the resistance to shear. Samples with significant halloysite amounts were more sensitive to drying shrinkage due to their morphology and high specific surface. Nevertheless, coexistence of quartz in the bulk composition of raw material balanced this parameter.

In 2009, Djambazov et al. [38] investigated white halloysite-rich clay deposits of the Kravelo village area (Bulgaria) derived from hydrothermal alteration of tuffiscite rocks. They were comprised mainly of halloysite, sanidine and minor of quartz and calcite. Despite the significant coarse grain’s participation in the matrix, their high air shrinkage and low mechanical strength on green, those clay raw materials were characterized by high %wt Al_2_O_3_ content (31–34 wt%) and appropriate plasticity, due to halloysite occurrence, and were proposed for the production of granitogres and clincer tiles and bricks. In the same year, Tezuka et al. [39] studied the impact of oxide additives (TiO_2_, Fe_2_O_3_) on the sintering processes in Alumina/clay ceramics derived from kaolinite or halloysite clay materials. In case of halloysite clay they suggested the oxides’ addition in order to improve the densification and cracking defaults derived from cristobalite existence. They indicated that the additives promoted sintering processes via the liquid phase and mullite formation in all samples. Moreover, similar mullite structures with titanium and iron ions in a solid solution were developed despite the different initial kaolinite or halloysite clay.

**Figure 3:**
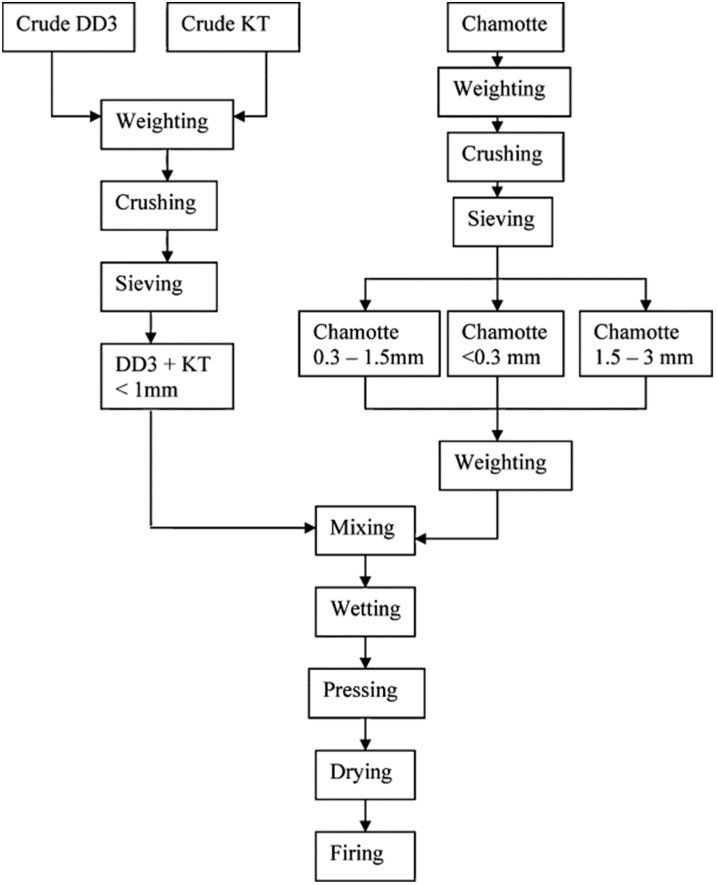
Schematic representation of the manufacturing process of the BSAA bricks [41].

**Figure 4:**
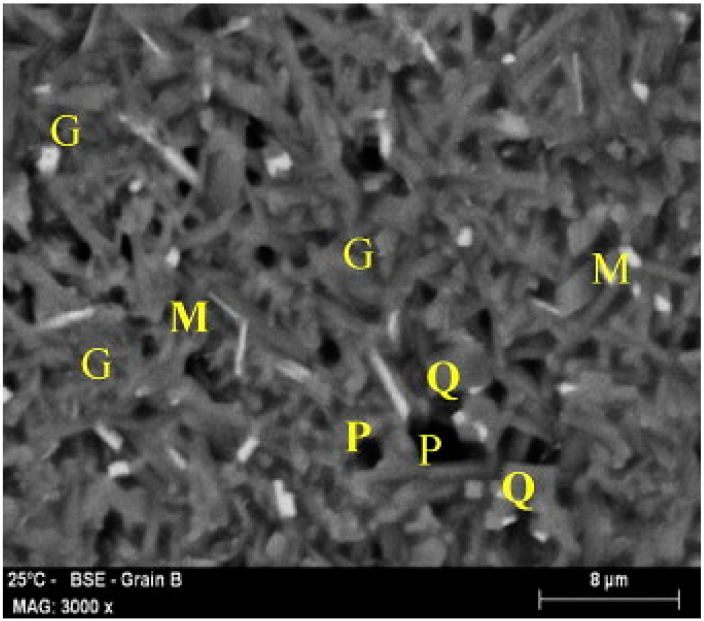
SEM micrograph of a BSAA refractory brick (halloysite and KT kaolin brick) fired at 1350 °C. M: mullite needles; Q: quartz; G: amorphous phase; P: pores [41].

O’Conor and MacKenzie [40], after dihydroxylation of halloysite clay, prepared lithium aluminosilicates compounds via the conventional method of geopolymer syntheses or by solid state thermal reactions of dehydroxylated halloysite with lithium hydroxide or lithium silicate. The new compounds consisted mainly of lithium zeolites, and minor amounts of quartz and cristobalite remained from the raw clay material. Zeolite crystals presented interlocking texture characteristics (similar to halloysite) contributing positively to the strength. The authors observed lower zeolite crystallization as the initial silica content increased since the pH values were increased, delaying the dehydroxilated halloysite dissolution. The next year, Amrane et al. [41] investigated the thermo–mechanical properties of new silica–alumina refractory materials derived from halloysite clay–kaolinite clay–chamotte and sintering up to 1350 °C (Figure 3 and Figure 4). Due to the fine particles (≤0.01 mm) and high plasticity of halloysite clay, the introduced chamotte and kaolinite clay in the starting mixture contributed mainly to the dimensional stability and appropriate physical properties of the sintered product, respectively. Authors highlighted the crucial parameter of the initial siliceous amount in clay material since transformations of high amounts of free silica promoted cracking during the sintering process and reduced the final mechanical properties. The same period characterization of halloysite-rich clay derived from N.Thailand was carried out by Bordeepong et al. [13], while other clays derived from the same region were also studied by Bordeepong et al. [42]. Those clays were classified as appropriate raw materials for ceramic production. In the case of halloysite-rich clays they consisted of tubular halloysite 7 Å, kaolinite, quartz and anatase, while after formamide treatment halloysite was calculated at 70 wt% in their bulk composition. Moreover, the heat treatment results indicated that, after 550 °C, the halloysite structure collapsed, while at 1001 °C the exothermic reaction of TGA/DTA curves was attributed to the initialization of mullite forming. Among the studied samples, kaolinite–halloysite clays were proposed as the best quality raw materials for white ware ceramic production, whilst halloysite-rich clays were suitable for porous synthesis with individual uses, e.g., fillers. Moreover, clay raw materials derived from N. Tunisia (Nefza region) were experimentally studied by Moussi et al. [43] for use in traditional ceramic industry. Among the studied clays fine kaolinite–halloysite ones exhibited high plasticity and high required moisture for shaping (58%), due to the halloysite nano tubular morphology and its high water absorption. These characteristics attributed the clay high drying and firing shrinkage (3.8% in Bigot curves and 8%, respectively) with the drying shrinkage value being further increased (4.5–6.1%) in semi-industrial scale tests. After mixing the aforementioned clays with other local raw materials containing quartz, feldspars and low phyllosilicates, the authors indicated that the processing and dimensional problems were reduced, and that the new products met the requirements for brick and tile production.

A few years later, Harabi et al. [44] produced low cost porous mullite-based ceramics with two different product shapes (via different processes (Table 1)) for their potential use as ceramic supports in membranes. Besides the halloysite clay raw material, they added calcite in order to form mullite and anorthite phases in the final products and avoid the undesirable cristobalite. According to the authors, the initial morphology of nano-rodes halloysite was a significant factor for the high mechanical strength of final products after firing at low relative temperatures: initial halloysite interlocked texture developed elongated micro crystals of mullite (starting at 994 °C) and anorthite forming a microstructure by necks or bridges.

Gass et al. [45] used starches (potato or corn) in accordance with (i) kaolinite rich-clay, talc and alumina or (ii) a commercial powder consisting of kaolinite, halloysite, talc, alumina and quartz in order to produce porous cordierite-based materials. The results show that after consolidation and sintering of samples, materials with halloysite exhibited higher mechanical strength compared to those with kaolinite. This was attributed to their advanced amounts of cordierite formation and glass phase derived from the low melting impurities presented in their initial raw material. Then, halloysite-rich clay from Ain Khemouda (W. Tunisia) was investigated by Jemai et al. [46] in order to be used in the ceramic industry. Raw material was characterized as aluminous zinciferous halloysite while in halloysite lattice iron participation was detected. According to the authors halloysite mineral indicated high amounts of interlayer water (halloysite 10 Å) and absorbed water in its predominant tubular crystals attributing 2% final drying shrinkage. Additionally, thermodilatometric analysis indicated dehydration of halloysite at 350–400 °C and satisfied firing shrinkage up to 1.4% at 850 °C, while mullite appearance was observed at 950 °C. The authors, taking into account these characteristics, established the initial mixture compositions and sintering profile in order to avoid defects during the forming and sintering processing of the new compounds. After firing of prepared mixtures of 95 wt% halloysite clay with 5 wt% dolomite up to 1050 °C, the preliminary results of technical tests revealed that they were appropriate for use as refractory bricks and earthenware tiles.

Afterwards, Senoussi et al. [47] collected and characterized clay materials from E. Algeria via different analytical methods. The authors confirmed the type of kaolinite group minerals based mainly on the formamide treatment raw material. They revealed that the studied clay consisted mainly of halloysite 10 Å (63–66 wt%) along with low amounts of other kaolinite group minerals, quartz, gibbsite and oxides, suitable for many ceramic and other nanotechnology applications. They stated the significant effect of flux oxide participation in bulk clay compositions during the sintering process and new formed ceramic phases. According to the authors, the plasticity and tear strength of the material was attributed to the predominant appearance of tubular halloysite crystals and to the tubular rods, respectively. Moreover, Zaiou et al. [48] developed successful anorthite-based ceramics with high density and anorthite content, after sintering of local Algerian halloysite-rich clay and pre-calcined calcite up to 1100 °C. Halloysite-rich clay was characterized by a homogeneous texture with low impurities and predominant nano-rod halloysite. The authors highlighted that the pre-calcination of halloysite-rich clay at 520 °C as well as the modifying milling of starting mixture had a positive effect on the sintering.

**Figure 5:**
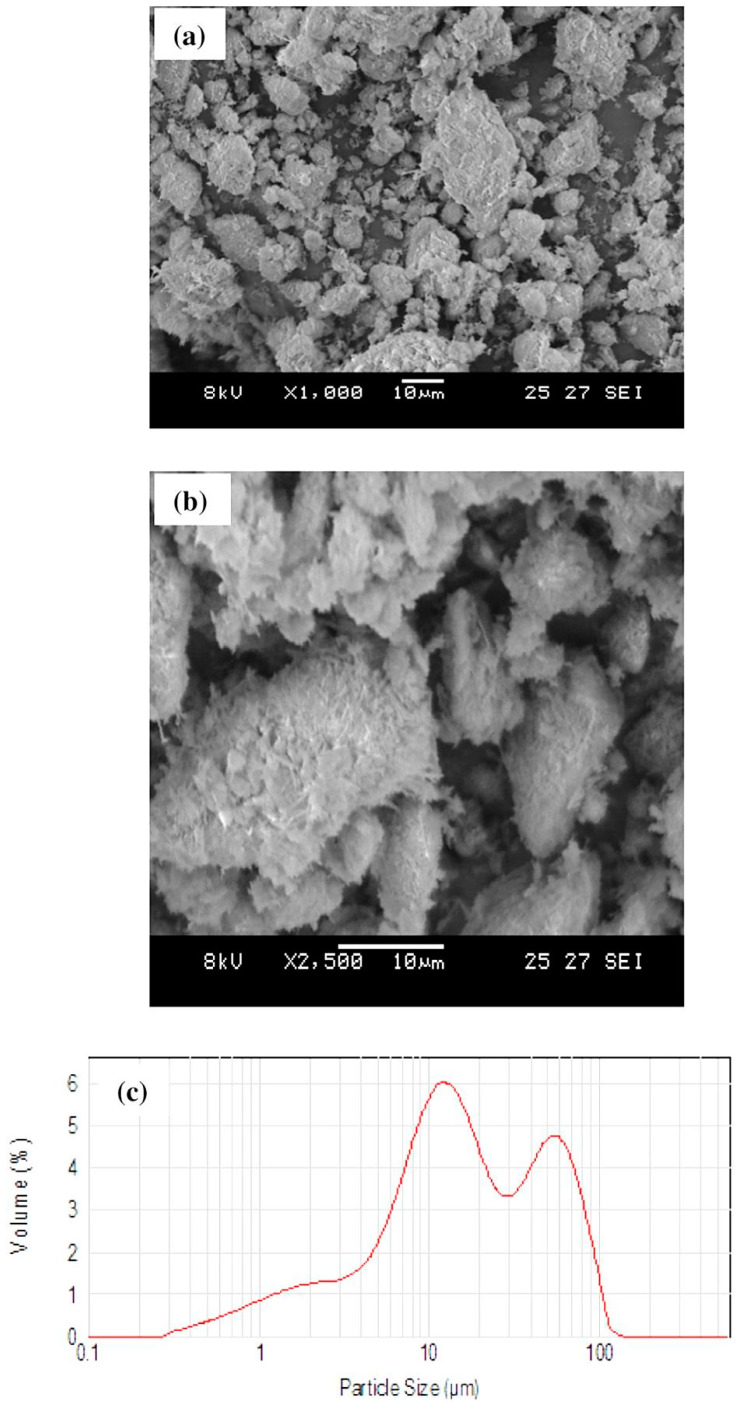
Raw halloysite, (**a**,**b**) SEM images, and (**c**) particle size distribution [50].

**Figure 6:**
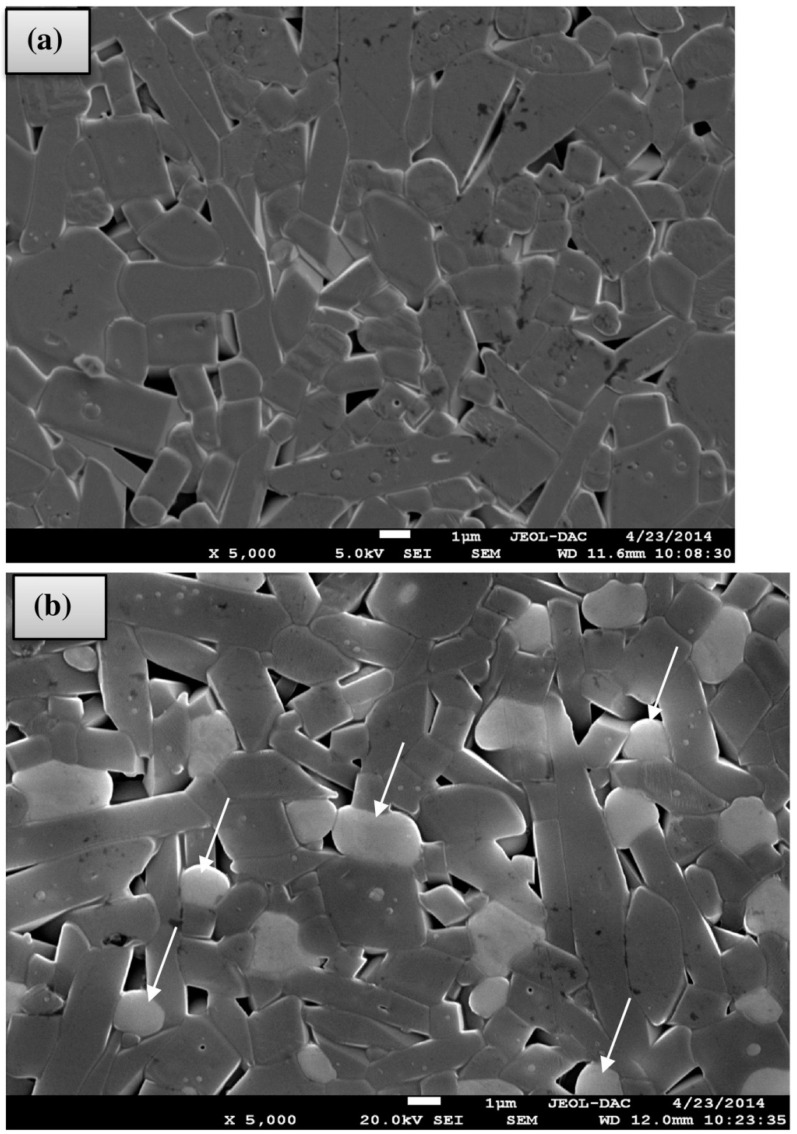
SEM images of surfaces of fractured samples sintered at 1600 °C for 2 h; (**a**) only the mullite phase in the halloysite–boehmite mixture and (**b**) the presence of both mullite and zirconia (see arrows) in halloysite–boehmite–zirconia mixtures [50].

The results of the characterization and potential uses of clay materials derived from different deposits of W.S. Sinai, Egypt was presented by El-Kammar et al. [49]. These materials were comprised mainly of kaolinite, smaller amounts of halloysite, dickite, quartz and minor amounts of smectite, illite, gypsum, dolomite, and hematite. The results show that, even though most of the studied samples contained the sought Al_2_O_3_ amounts, they were convenient for international ceramic production only after a beneficiation, removing the excess amounts of iron and titanium oxides. In the same period, Raghdi et al. [50] developed low cost mullite–zirconia ceramics after sintering up to 1650 °C of different mixtures of Algerian halloysite clay raw material, boehmite and zirconia. Despite halloysite being usually presented with nano-tubular morphology, in this study platy or sub euhedral crystals of halloysite 10 Å with particle sizes ~10–50 μm were observed in the matrix (Figure 5). Thermal analysis of halloysite-rich clay indicated dehydration of halloysite at 556 °C and primary mullite formation at 1171 °C. Additionally, the prepared ceramic compositions exhibited similar thermal behavior, detecting metalloysite (meaning halloysite 7 Å) and mullite at 546 and 1153 °C, respectively. According to the authors, the aforementioned results revealed that the phase transformations during the sintering processing depended mainly on halloysite transformations, while additive enhanced the microstructure strength further (Figure 6).

A year later, Bouzidi et al. [51] added ΒaCO_3_ in to kaolinite–halloysite clay in order to promote sintering process and to increase the refractory index of final ceramic compositions, produced after firing at 1100 and 1200 °C. The authors observed decrease in the dehydroxylation temperature of halloysite (from 501 to 494 °C) with the increase in ΒaCO_3_ amounts (0–60 wt%). Moreover, the initial mechanical activation of raw material promoted the reaction kinetics during the sintering since destroyed halloysite and kaolinite became more reactive. According to their results the refractory phases of hexaselcian (barium aluminosilicates) were formed by the addition of ΒaCO_3_ up to 50 wt% both at 1100 and 1200 °C. Haddar et al. [52] studied Morroccan halloysite-rich clays (Nador, NE Morocco) on the base of their suitability for ceramic production. They indicated that the hallοysite raw material consisted of halloysite 7 Å, gibbsite, alunite, K-feldspar and minor smectite, illite minerals. The high plasticity (31.5%) and high specific surface area of raw clay material was attributed mainly to the tubular morphology of halloysite and its high amounts of bounded water, as well as to the absence of quartz. According to their thermal analysis from 500 to 1100 °C, decomposition of halloysite led to the new phases of mullite (at 975 °C) and spinel. After mullitization (development of cuboidal and needle crystals of mullite), the final products exhibited advanced flexular strength (16.3 MPa). According to the authors, the studied clays were suitable for refractory production, whilst the addition of quartz was proposed in order to reduce the plasticity and cracking during the sintering process. In the same year, Sanz et al. [53] investigated the effect of two different kaolin raw materials (kaolinite-rich clay and halloysite-rich clay) on the final whiteware porcelain products. They demonstrated that, despite the similar chemical compositions of the two starting clay materials, the morphological characteristics and crystal size of clay minerals of kaolinite and halloysite, along with the heating rate, influenced the mullite crystallization in the sintered materials. Among their results, they indicated that final porcelains contained similar mullite composition and content whilst, under the same temperature and heating rate, mullite derived from halloysite exhibited higher values of particle crystalline size.

Moroccan halloysite-rich clay with a high content of aluminum was the major raw material for the production of new silico-aluminous refractory products after firing at 1300 and 1500 °C by Haddar et al. [54]. Due to the high shrinkage of halloysite, it was mixed with local diatomite, marl and silica sand, improving the cracking phenomena during the sintering. In a second stage, 25% addition of recycled alumina (silico-aluminous refractory brick waste) in the aforementioned mixture and sintering at 1500 °C further improved the mechanical properties of the new ceramic, especially its flexular strength value (45.08 MPa), due mainly to the high content of mullite which occurred in the refractory waste and its elongated crystal shape. At that time, because of the significant reduction in the kaolin deposits in Korea used in porcelain production, Kim and Hwang [55] investigated alternative clays derived from China and Vietnam. They indicated that the whole raw materials studied (Korea, China, Vietnam) were comprised mainly of kaolinite and muscovite and less of halloysite, quartz and rutile. Higher amounts of halloysite (up ~7 wt%) were found in samples derived from China and less in those in from Korea. The new deposits have lower iron content (<0.5%), exhibiting higher whiteness and characterized by a uniform particle size distribution in their matrix. Moreover, samples derived from China and Vietnam exhibited suitable plasticity due to the planar and needle-like shape crystals of clay minerals. Lao et al. [56] produced cordierite ceramic materials suitable for thermal storage, after adding MgO in the starting mixture of halloysite-rich clay, calcined talc, α-Al_2_O_3_ and sintering up to 1400 °C. According to the authors, in case of cordierite ceramic production, tubular halloysite crystals in raw material affect powder flowability positively but remaining highly elongated tubular halloysite at high temperature inhibited the viscous flow and further densification, demanding higher temperatures or liquid content in their matrix. They indicated that 0.5 wt% MgO addition affected the full dissolution of tubular halloysite in the matrix during the sintering, enhancing the cordierite crystallinity and decrease in pores along with increase in the densification and heat capacity of the final product. Moreover, among the results, the authors highlighted the thermal heating control of time at 300 and 500 °C in order to avoid defects during the halloysite 10 Å transformations. Additionally, the final properties of porcelain products derived from mixtures of Algerian halloysite-rich raw materials and ZrO_2_ additive after sintering at 1000–1250 °C were investigated by Serragdj et al. [57]. The new products consisted of mullite, quartz and glass phases exhibiting increased flexural strength and microhardness compared to that of the conventional porcelains.

Recently, Cheraitia et al. [58] developed cost effective mullite–cordierite refractories from kaolin, fireclay and talc raw materials. They studied the substitution of the French imported kaolinite-rich clay material with the local halloysite-rich clay with higher aluminum in the refractory production. According to their results, the final sintered samples with halloysite-rich clay exhibited higher contents of cordierite and mullite and better thermomechanical characteristics due to the more plastic- and aluminum-rich halloysite clay. Moreover, fireclay (consisting mainly of mullite and sillimanite) was added in order to improve the physical properties of the initial mixtures as well as the refractoriness of the products, while talc offered magnesium for the cordierite formation.

#### Overview and Discussion

Most previous investigations on halloysite-rich clays focused on their bulk chemical–mineralogical compositions and ceramic properties in accordance with different additives used in many cases. The results revealed that the use of halloysite-rich clays derived mainly from Asia, Africa and Oceania (only N. Zealand) and less from Europe (Table 1).

Compositions of such materials were varied, strongly influencing the sintering processing and their potential use, covering a broad field of applications as refractories, porcelain, tiles, earthenware products, etc. Generally, clays with halloysite consisted of halloysite, other clay minerals, feldspars, quartz and minor oxides. In most of the cases, the major phases of final products were mullite and/or other refractory phases (e.g., cordierite). Plasticity, rheology, shaping and sintering processing are some critical parameters which were referenced that were strongly affected by the presence of halloysite.

Despite this, halloysite characteristics (amount in raw clay material, morphology, microchemistry, type, thermal behavior) were partially or not provided in the literature but when this did occur, very important information about the effect of halloysite mineral on traditional ceramic production was given and it is discussed in the following paragraphs. Moreover, the kaolinite group minerals were scarcely discriminated by IR or formamide testing [13,43,44]. We believe that, in order to investigate the effect of halloysite presence, quantitative analyses should be provided, coupled with a thorough discrimination of kaolinite group minerals using IR and the formamide testing when needed [59]. In some cases, this discrimination was based on the crystal morphology, which is not always correct [60].

- The effect of halloysite-clay raw material composition and halloysite content in the formation and firing processing.

Among studies in which clay raw material’s quantitative mineralogical results were provided, the halloysite amount varied over a wide range (7–90 wt%), with most of cases detected up to 40 wt% and only a few <20 wt%. Despite of these differentiations, the majority of these studies focused on the same direction of mullite-based ceramic production and less other refractories after firing in most cases up to ~1350 °C. The increased halloysite content affected the final mullite content increase, starting to be formed at ~950–1170 °C. Higher amounts of clay raw material with low percentages of halloysite could be used in the prepared initial mixtures compared to halloysite-rich clays with higher contents of halloysite, in order to produce acceptable ceramics (e.g., [46,54]). Moreover, among different clay raw materials, those with higher contents of halloysite exhibited higher plasticity as well drying shrinkage (e.g., [37]) due to predominant tubular morphology of halloysite with its small particle size and water content. Indeed, nanotubes were the most common crystal shape of halloysite in raw clays, usually contributing positively to the formation processing. Nevertheless, cracking phenomena could develop due to the high dry shrinkage, especially in clay raw materials which lack non-plastic and coarse grains (e.g., quartz, feldspars) and halloysite ranges in high amounts. For this reason, many researchers proposed the addition of other materials, mainly siliceous ones, into the initial mixtures (e.g., [41,43]), which also controlled the firing shrinkage. Moreover, impurities in raw clay materials (e.g., oxides) acted mainly as fluxes enhancing liquid phase appearance at lower temperatures and halloysite reactivity. Obviously, their amounts influenced the final properties of the ceramic and its uses. For instance, in the case of porcelains, iron oxides did not exceed the 1–2 wt% of bulk chemical composition (e.g., Senoussi et al., 2016). Additionally selective additives promoted sintering and densification, further increased the new refractory phases and the final strength, minimized shrinkage or prohibited other cracking phenomena derived from quartz or cristobalite transformations during the firing processing (e.g., [39,51]).

Halloysite and kaolinite coexistence in kaolinite–halloysite clay raw materials exhibited a good synergistic effect in the formation and firing processing. In cases of significant amounts of tubular halloysite and its high plasticity, kaolinite balanced dimensional problems (e.g., [41]). Despite a complex of parameters was involved during the sintering, investigations revealed similar mullitization degrees of kaolinite rich clays and halloysite rich clays in ceramic production (e.g., [34,53]). Nevertheless, due to the smaller halloysite particle size and tubular morphology, halloysite-rich clays could be more reactive, favoring the crystallization [53].

Despite the fact that the previous investigations considered the introduction of different mineralogical additives in the starting raw material, studies on valorization of halloysite wastes as well as other low-cost industrial byproducts in traditional ceramic production are still in an initial stage, although they could be very promising [61]. The researcher’s interest should probably be focused to the improved processes and final products with further environmental and economic benefits, promoting simultaneously the global demands of the circular and green economy. For example, this could be the case in localities where mining halloysite raw deposits generates large amounts of solid sterile wastes and/or other secondary industrial materials are provided nearby.

- The effect of halloysite type and structural characteristics on formation and firing processing.

Disordered halloysite or impurities in its lattice, its low particle size (nanocrystals) and high surface area promoted its reactivity and consequent mullitization and refractoriness during the heating treatment [28,36,39,46]. Moreover, the interlocked tubular halloysite crystals of raw material suggested that it could increase the strength of final products: halloysite retained its primary morphology at high temperatures and produced elongated mullite crystals which also exhibited a similar interlocking texture [44]. It was difficult compare the different halloysite morphologies’ effects in ceramics since only in a published work [50] were they detected in a plate and not tubular morphology. However, slightly higher temperatures of halloysite dehydroxylation (556 °C) compared to those in other referred studies (e.g., 520, 501, or 534 °C 48, 51, 52, respectively) were observed. This could be attributed to the different surface area and higher particle size of plate halloysite (10–50 μm), which probably reduced the kinetics.

The initial halloysite type (10 or 7 Å) was provided in some works. This is important information since it could affect shaping or heating processing but extended correlations are lacking in the literature. Nevertheless, Lao et al. [56] gave special attention to the thermal analyses of halloysite clay and specifically to the exact dehydration temperatures of halloysite 10 Å in order to avoid failures during the sintering of ceramics. According to the results of Senoussi et al. [48] and Raghdi et al. [50], the halloysite 10 Å transformed to 7 Å at temperatures up to 400 °C regardless of its different morphology of tubular or plate crystals

### 4.2. Advanced Ceramics

**Figure 7:**
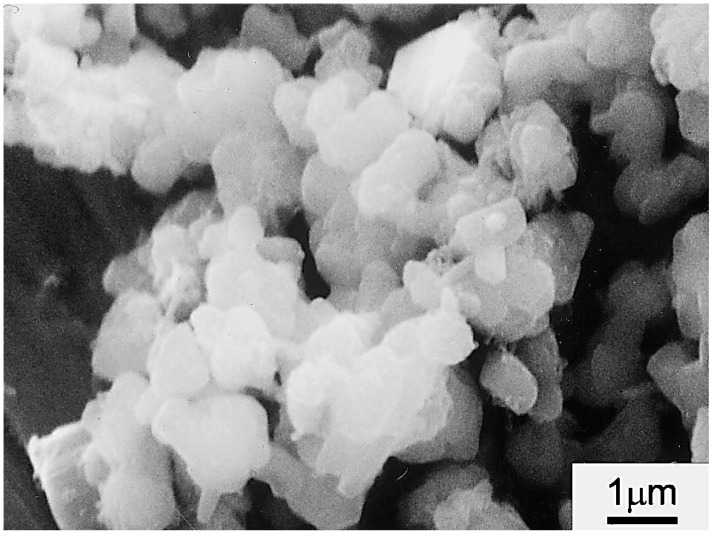
Representative SEM image of a SiAlON microstructure, synthesized from talc and halloysite [62].

The most commonly used advanced ceramics syntheses are the SiAlONS. They constitute syntheses on the system Si-Al-O-N that consists of silicon–aluminiumoxinitrides. They have extensive uses in many engineering applications, especially in those involving high temperatures, because they exhibit advanced thermo–mechanical properties and corrosion resistance. Various types of SiAlON (as α,α’, β, β’Χ, O) with different chemical and structural characteristics and properties are usually synthesized from mixing raw materials such as Si_3_N_4_, AlN, Al_2_O_3_, with several additives after firing at high temperatures. Nevertheless, their use on the industrial scale was usually restricted due to the highly expensive raw materials and processing procedures [62]. As a consequence, many researchers had focused their investigations on the utilization of low-cost raw materials and more simple treatments for the production of SiAlON compounds. Hence, more versatile methods of SiAlON were introduced such as carbothermal and nitridation (CRN) or silicothermal methods, which utilize inexpensive and abundant clay minerals as staring materials (Table 2). They were based on the initial easy thermal decomposition of clay minerals to mullite and silica, which were subsequently involved in a series of intermediate reactions in the presence of carbon and nitrogen or only nitrogen (Figure 7). The properties of the final products were influenced strongly by several parameters such as composition, mixture preparation, time and temperature of processing, additives, etc.

In 1976 Lee et al. [63] presented the silicothermal method as a relatively low cost and easy treatment for SiAlONS production. They used kaolinite or halloysite and carbon as starting materials and firing in an oxidizing temperature up to 1800 °C, developing promising products with equal properties.

Almost 18 years later, Μackenzie et al. [64] studied the mineralogical and structural characteristics, during the intermediate and final reactions stages of carbothermal synthesis of β’-SiAlONS derived from halloysite or kaolinite and carbon, using combined XRD and NMR techniques. XRD and NMR results agree with the reaction sequence, whilst they provided complementary information as regards the phases transformations mechanism during the firing, up to the maximum temperature of 1400 °C. Both in kaolinite and halloysite clays was mullite crystallization initialized at 1100 °C. Nevertheless, they revealed that the cristobalite phase, presented only in halloysite samples, delay the formation of the intermediate SiC compared to that in kaolinite ones. At the same time period, and in a similar way Neal et al. [65], produced β-SiAlON by carbothermal reduction and nitridation from halloysite clay at 1400 °C, and they used several techniques to determine the phase characteristics and transformations during the firing. The succeed final product was characterized by the chemical formula of Si_6_-zAlzOzN_8_-z; 0 < z < 4.2.

Μackenzie et al. [66] proposed a carbothermal formation of inexpensive low-z β’-SiAlON compounds (z = 0.5) from SiO_2_/halloysite clay or Si/halloysite clay raw materials and Y_2_O_3_ as additive. They indicated that, during the formation of final low-z ceramics from SiO_2_/halloysite mixtures, similar reactions took place compared to those on SiAlONS with a higher z value (2.5–3). In the case of mixtures with Si/halloysite clay, O’-SiALON in combination with β’-SiALON phases was produced. The addition of Y_2_O_3_ to the starting mixtures enhanced the decomposition of halloysite and mullitization via the liquid phase at lower temperatures (<1150 °C), while it promoted the reactions at higher temperatures up to 1400 °C. Consequently, Ektsrom et al. [67] produced CRN α-SiAlON precursor powders and final dense ceramics from halloysite clay as a major raw material. According to their study, the undesirable high Al_2_O_3_/SiO_2_ ratio of clay material was balanced by the addition of SiO_2_ or elemental Si to the starting mixture, in order to promote sintering. The reactions of halloysite to mullite and amorphous phase transformation were mainly dependent on temperatures and were carried out at intermediate ones. They revealed that the appropriate elemental Si (instead of SiO_2_) facilitated the reactions at low temperatures and restricted the amorphous SiC. Moreover, the high Y_2_O_3_ amount in the starting mixtures contributed to the densification of final ceramic products.

Sheppard and MacKenzie [68] studied the influence of 1 wt% metal oxide additives on the silico–thermal formation of X-SiAlONS (solid solution of mullite-Si_3_N_4_) at 1200–1500 °C from a starting mixture of halloysite clay, alumina and elemental Si. They referred that silica derived even from the initial mineral composition of hal-loysite (as quartz) either after the clay decomposition, promoted the formation of the intermediate silicon oxinitride phases and consequent final X-SiAlONS. Moreover, they indicated that the additives of Y_2_O_3_, CaO, CeO_2_, and to a lesser extent MgO, favor the mechanism of reactions and densification of final X-SiAlON formation. After this published study, Zhang et al. [62] synthesized Mg-αSiAlONS powders from low cost halloysite clay and talc raw materials via a carbothermal reduction and nitridation method. The final products consisted of the major α’ and β’ phases. The magnesium rich raw materials of talc positively affected the cooperation with halloysite and final densification and uniform morphology of new synthesis.

Two years later, Qiu et al. [69] published the results of a CRN Mg-αSiAlON synthesis from halloysite clay and talc minerals too. They investigated several parameters during processing such as carbon content, temperature and starting mixture and stated that the final products are strongly influenced by these parameters. They concluded that the most effective conditions for the new synthesis consisting of 90% Mg-α SiAlON phase and uniform morphology were those of a halloysite/talc ratio equal to 1.5/2 and firing at 1480 °C for 4 h. Additionally a molecular ratio 1.00 of carbon was the appropriate amount, since higher contents prevented the nitrogen penetration to the clay surfaces and reaction progress.

**Figure 8:**
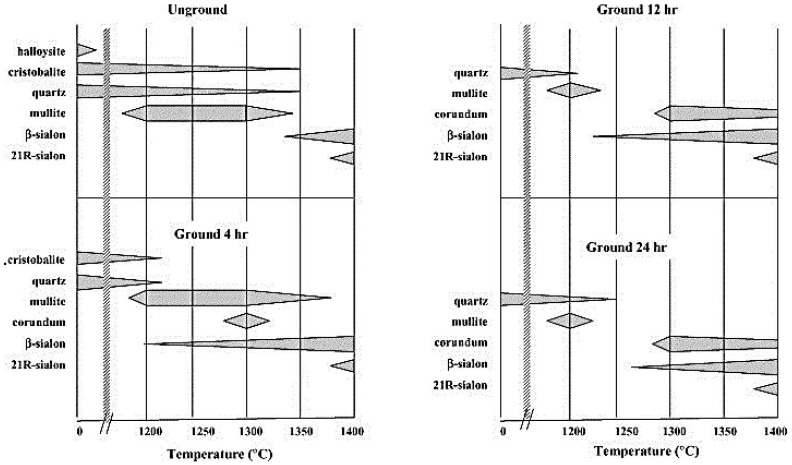
Schematic representation of the crystalline phases formed from the ground and unground CRN precursors in [1].

After four years, MacKenzie et al. [1] investigated the effect of the mechano–chemical activation of starting mixture on the final CRN β-SiAlON product. The results show that 12 h grinding of the starting mixture promoted the reactions during the firing process and SiALON formation. Moreover, they demonstrated that halloysite decomposed in ground mixtures after 4 h grinding. Eventually, they concluded that the unground mixtures demanded temperatures of 1300–1400 °C in order to be transformed to the final SiAlON products. On the contrary, in case of the ground mixtures, the intermediate phases disappeared earlier, and SiAlONS were formed at <1300 °C (Figure 8).

After a significant time-gap of investigations on this topic, Yin and Jones [70] synthesized SiAlONS with z values of one and four and analyzed the intermediate phases during firing at various temperatures. They indicated that, as the temperature was increased, the phases transformations to β-SiAlONs were increased, as well as their crystal size with the optimum firing temperature at 1400 °C.

Beyond the aforementioned, in the national field have been presented some other significant published investigations on SiAlON synthesis from kaolin as the starting material [71,72,73,74,75], which probably consist of both kaolinite and halloysite minerals. In these studies, the specific mineral compositions of kaolin raw clay were not clearly presented, and for this reason they have not been included in Table 2.

#### Overview and Discussion

The total previous investigations indicated that SiAlON products, derived from halloysite clays, are promising materials due to their final properties In most cases the halloysite raw material was derived from China or N. Zealand (Table 2).

Mixing of halloysite clays with other low-cost additives further enhanced the halloysite decomposition and densification during the firing processing and produced successful SiAlONS, after carbothermal or sometimes silicothermal treatment in a nitrogen atmosphere at about 1400–1500 °C. Researchers used halloysite–rich clays which easily decomposed and reacted to offer the desired intermediate mullite phase. However, SiAlON products derived from halloysite seem not to be in the spotlight of research for the last 20 years and this is not a coincidence. Despite this, a new innovative technology that could provide the synthesis of a more efficient or cheaper material cannot be excluded. Moreover, although the published articles provided the bulk chemical compositions of halloysite-rich clays, no further particular characteristics of halloysite mineral and effects (e.g., morphology, particle size) or bulk mineral composition are provided. This is a scientific gap in the investigations since a such characterization could favor further processing and total production cost, e.g., avoiding clays with high impurities and low amounts of halloysite, applying an easy pre-beneficiation or other mechanical activation of raw material, forecasting synergy of additives as well as improving sintering profiles.

### 4.3. Ceramic Composites

The term “ceramic composites” will be used in this study for smart complex halloysite-based synthesis, described in the literature as materials with some ceramic properties and specific applications (e.g., coating, membranes—Table 3).

Fu et al. [76] and Fu and Zhang [77] fabricated metallized-ceramic microstructures with magnetic properties using halloysite as the template substrate. Initially, Pd nanoparticles activated the halloysite for the consequent Ni plating and heat-treatment at 400 °C. According to Fu et al. [76], the heated metallized ceramic materials exhibited higher saturation magnetization compared to that in its as-plated form. In the case of Fu and Zhang [78] Ni nanoparticles and wires were deposited simultaneously on the halloysite outer surfaces and inner cavities, respectively. They concluded that the higher amounts of iron impurities of halloysite in its inner surfaces enhanced the Pd reduction in these sites increasing the electroless Ni wire deposition. In the same period, Byrne and Deasy [78] produced pellets of porous aluminosilicate ceramics for drug delivery using halloysite or kaolinite. The higher amounts of the drug were precipitated in the halloysite compared to those in kaolinite, due to the microtubules of this mineral. Five years later, Forsgren et al. [79] also produced a ceramic drug delivery carrier after pelletization of halloysite with fine cellulose. Their drug release analyses based on the Weibull equation and their preliminary results show that the final studied ceramic vehicle could be investigated further in the future as a promising composite for new opioids.

Four years later, Fakhrullin et al. [80] prepared ceramic–polymer composites with anticorrosion inhibitor-loaded halloysite used as coatings for different alloys. They revealed that 5% halloysite in the polymer composites increased their mechanical properties (bending modulus, tensile strength) and resistance to ignition due to the new ceramic frame of halloysite tubes in the polymer matrix. Moreover, their improved anticorrosion properties were attributed to the slow release of corrosion inhibitors from the halloysite to the coating defects, limiting the corrosion in an initial stage. Consequently, Vinokurov et al. [81] fabricated a promising cheap Al–halloysite substrate coated with metals by vacuum evaporation in order to improve the SERS (surface enhanced Raman spectroscopy) activity.

Chen et al. [82] produced low-cost halloysite-based ceramic nanofiber mats by electrospinning and sintering for use as support in zeolite membranes. The new ceramic materials were characterized by higher interconnected porosity, mechanical strength, flux and adhesion compared to the a-Al_2_O_3_ commercial support of zeolite. They also exhibited that the external silica layer of halloysite nanotubes prevented Al migration to zeolite. Kujawa et al.’s [83] manufactured porous halloysite based-ceramic performs well for novel applications in metal matrix synthesis. The results show that, during the sintering of halloysite nanotubes with carbon fibers at 1500 °C, the bonding strength increased as the halloysite content increased (up to 70%). Nevertheless, they concluded that more investigation was needed in order to be obtain materials with open porosity ranges from 60 to 85%. Pawlyta et al. [84] produced porous mullite ceramic preforms from halloysite and carbon fibers after sintering at 1300 °C, consisting of mullite and cristobalite. Consequently, the mullite ceramics were infiltrated with porous aluminum alloy filling. The final product exhibited a metal skeleton in a dense ceramic matrix of mullite, while the cristobalite was decomposed. Moreover, a transition zone of γ-Al_2_O_3_ and spinel between the metal and ceramic textures was developed.

Ghaderi-Ghahfarrokhi et al. [85] elaborated nanosyntheses consisting of polymer and HNTS. They indicated that modified HNT (HNT-NH_2_, HNT-COOH) exhibited improved distribution on a PCL (polycaprolactone) electrospun substrate and intercalated nanostructures. Consequently, the ceramic characteristics of modified HNTS nanocomposites improved their mechanical and thermal properties compared to those with neat HNTS. Additionally, the modified HNTS contributed to the drug absorption capacity due to the increased electrostatic interactions. Vinokurov et al. [86] suggested halloysite as a cheap, environmentally friendly mineral suitable for the easy production of ceramic core–shell materials by Schiff base binding, in order to be used for metal nanocatalysts or metal adsorbent from solutions. They indicated that furfuraldehyde-loaded halloysite was transformed to efficient ligands for increased binding of metal ions. Moreover, the reduction and cooling of these composites contributed further to the higher metal particle concentrations on the outer and inside of halloysite tubes.

In 2018 Guo et al. [3] studied the ceramizable nature of silicone rubber (MVQ)/halloysite (HNT) composites with different borate additives (sodium tetraborate decahydrate, ammonium pentaborate, zinc borate) as well as their ceramic residues after firing at 1000 °C. They revealed that halloysite and zinc borate reaction during the sintering of the sample led to the formation of the new phases of mullite and gahnine which contributed to the increase in flexular and impact strength of the residues. At the same time, the aim of the Zhang’s et al. [87] work was the utilization of solid wastes consisting mainly of halloysite and iron oxides (low grade pyrite cinder, LPC) for the production of magnetic ceramics. After microwave heating of LPC with coal powder and low amounts of calcium fluoride, the final ceramic products were characterized by the major phases of mullite and magnetite in an interconnected microstructure, attributing it advanced compressive strength. Moreover, they indicated that the new ceramics could be used as aggregates in asphaltic mixtures with deicing properties in cold climates.

**Figure 9:**
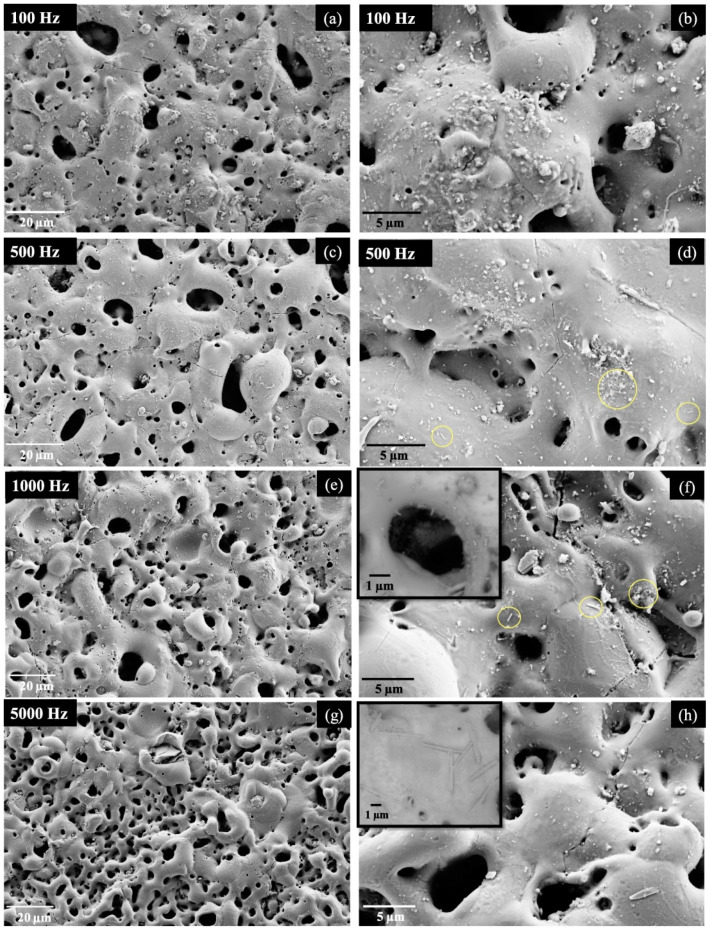
SEM micrographs of HNT-containing PEO coatings produced at (**a**,**b**) 100 Hz, (**c**,**d**) 500 Hz (**e**,**f**) 1000 Hz and (**g**,**h**) 5000 Hz frequencies. Yellow circles indicate remaining HNTS [88].

The results of integrated HNTS as nanocontainers of chemical active agents in forsterite PEO-obtained coating on an AM50 alloy were presented by Mingo et al. [88]. They indicated that HNTS modification is strongly influenced by the thermodynamic conditions of the surface. At pulses longer than 10–4 s (low frequencies) and 950 °C, the surface was heater-activated by the alloy substrate, increasing the crystallization of forsterite and decomposition of HNTS. At short pulses (2 × 10^−5^ s, 5000 Hz) grain growth of forsterite was not pronounced and HNTS retained their tubular form (Figure 9). They concluded that observations of the coating microstructure in accordance with thermodynamic surface conditions could be provide coating with specific demands and properties. Recently, another investigation on coating materials with halloysite was provided in the literature from the Molaei et al. [89]. They developed chitosan–ceramic nanocomposite coatings on a Ti-substrate by an electrophoretic deposition method (EPD). They fabricated multi-component coatings consisting of chitosan (CS), bioglass (BG), hydroxyapatite (HA) and halloysite nanotubes (HNT) and studied several process parameters such as deposition time and voltages, the insulation process, the effect of components, etc. Among their results they concluded that HNTS contributed to the improvement of the mechanical properties of coatings while halloysite delayed the ions’ diffusion and consequent corrosion of the substrate. Petrova et al. [90] produced metal–halloysite ceramic syntheses (Pd, Pt/HNTS) used in perfluorinated hybrid membranes. The final membranes exhibited improved thermo–mechanical, electro-transport and selectivity properties demanded by fuel cell engineering and electromembrane processes. Halloysite nanotubes, due to their hydrophilic and polar characteristics, enhanced the ions exchanges as well as the moisture of membranes. Moreover, Pd, Pt doped metals acted as efficient catalysts in the modification of membrane characteristics.

**Figure 10:**
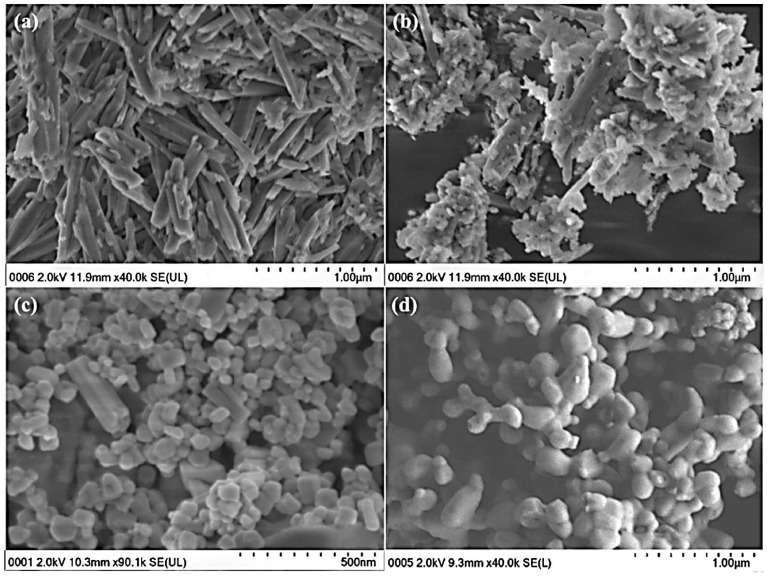
SEM Microphotographs of (**a**) pure HNT morphology, (**b**) acid-treated HNT forming SiNP, (**c**) nanoparticles of Clay-NZS, and (**d**) micrograins of Synth-NZS [91].

Johari et al. [91] synthesized clay-based Na_2_ZnSiO_4_ (Clay-NZS) materials using silica nanoparticles (SiNP) as a starting material. SiNP was derived from sulfuric acid treatment of halloysite nanotubes and characterized by smaller sizes (50–70 nm) compared to the synthetic ones (200 nm), contributing to a denser structure and higher conductivity (Figure 10). Moreover, low amounts of Al impurities remained after halloysite treatment, doped in the final compounds, further raising the conductivity. Recently, Mingo et al. [92] produced ceramic coatings by plasma electrolytic oxidation (PEO). Different corrosions were incorporated into halloysite nanotubes of PEO coatings. After that, they managed to control the release of active agents under pH changes and consequent corrosion of the metal substrate due to the high strength properties of halloysite-based ceramic coating in combination with the self-healing properties of inhibitors.

#### Overview and Discussion

According to the abovementioned studies, the interest of scientists and investigators in ceramic compounds with HNTS have increased, especially in recent years. Halloysite has been the ceramic material used in several fields of smart applications due to its tubular morphology, high specific surface area as well as its different inner and outer chemical properties and environmentally friendly character. Moreover, it is abundant and inexpensive compared with other nanotubes materials. In most of cases high purity HNTS, the raw material used in ceramic compositions is derived from N. Zealand, China and U.S.A. deposits (Table 3), whilst its treatments depend on the final product and use (Table 3). For instance, heating processing is performed in cases of final metallic–ceramic materials, porous ceramics or other ceramizable materials. Plasma electrolytic oxidation or other deposition treatments were applied for the synthesis of coatings or substrates. In case of HNTS-based synthesis used as a drug delivery vehicle, pelletization processing has been performed in different investigations.

A lot of previous investigations are concerned with polymer–halloysite-based ceramic materials for several uses, e.g., nanocontainers–carriers, ceramic coatings, ceramic membranes. The results indicated the good synergistic effect of polymers with HNTS in different ceramic materials synthesized through several methods (Table 3). Moreover, halloysite in most cases enhanced the thermo–mechanical properties due to the development of a ceramic skeleton in the matrix, or acted as a carrier and release adjustment for drugs or other chemical agents, e.g., anticorrosion inhibitors [19].

Halloysite has been used in metallized-ceramic materials with magnetic properties, while less ceramic products have been proposed for use as fire resistant materials, aggregates, electrolytes or substrates. Additionally, in many cases modification of halloysite led to the improved efficiency of its final properties due to the better deposition or loading capacity produced; HNTS were successfully involved in a broad field of semi-ceramic applications and markets, especially in high-tech products or medicines (e.g., [91]). This field is rapidly developing and therefore the studies on low-cost HNTS uses in advanced ceramic composites are sure to be enhanced in the future. Since the variety of such new products and uses is dedicated to very crucial and sensitive fields (e.g., for pharmaceutical products), basic economic analyses could be very helpful for a visualization of their scale-up capability, but they were absent in the literature.

## 5. Scientific Interest, Trends and Perspectives

**Figure 11:**
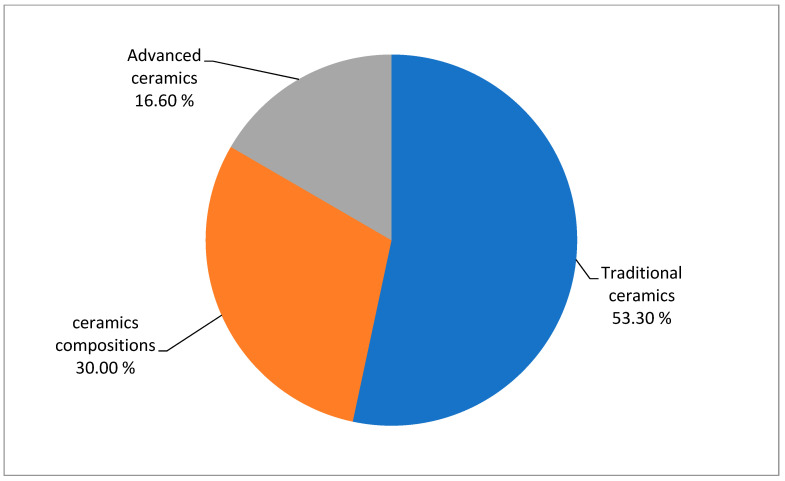
**%** Participation of the literature (of 55 references) on the different halloysite-based ceramics of the current review.

**Figure 12:**
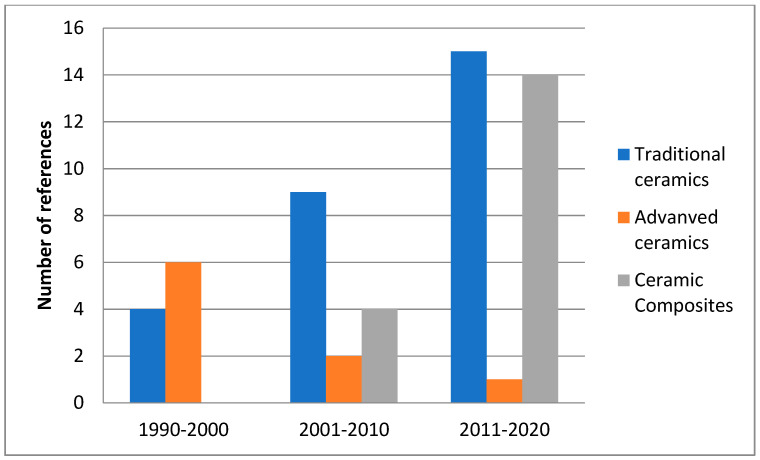
Scientific interest in time on different halloysite-based ceramics of the current review.

The scientific interest the last three decades on different halloysite-based ceramics and occurring trends can be visualized in the quantified diagrams of Figure 11 and Figure 12. Figure 11 clearly depicts the high scientific interest in traditional ceramics and ceramic composites based on halloysite, which covers the 80.3% among the total referenced literatures. On the other hand, significantly fewer studies have been published on advanced ceramics with halloysite raw material (16.6%). Figure 12 shows that there is a steady interest in time with a positive trend revealed in traditional ceramics and a clear upward trend in ceramic composites, especially in the last decade, whereas it is clear that research concerning advanced ceramics has been almost abandoned.

In most cases, investigations of this review revealed promising results for ceramic materials, allowing definition of technological development. Despite this, most studies are based on lab-scale experiments and only one paper regarding traditional ceramics referred to semi-industrial investigation [43]. This constitutes a disadvantage, and it is suggested that it would be useful for new investigations to be focused on pilot or large-scale applications, that way further increasing the interest of the ceramics industries in new developments, especially nowadays when the global demands for new ceramics products are only increasing, along with the competition.

In the case of traditional ceramics, full characterization of halloysite minerals in raw clay materials is an interesting and necessary topic and has not yet been extensively investigated. We believe that future studies could focus on that, since it could explain or predict material’s behavior or defects during the formation and sintering processing [93,94], especially under upscaling processing. Additionally, due to the significant fast rate of increase in research on new high-tech halloysite-based ceramic composites, they could be accompanied with basic economic analyses in the future, something which did not exist in the published papers. This will help further in upscaling of the experiments and establishing a go-to market strategy in this broad field. Regarding the advanced ceramics, the review revealed that detailed studies on halloysite clays’ characteristics in accordance with investment on new technologies are requested in order to continue relative investigations.

## 6. Conclusions

The present work constitutes an effort to sum and review previous publications concerning halloysite-based ceramic materials and “ceramic composites”. Halloysite-based raw materials cover a broad field of ceramic applications and demands with a constant interest on the exploration of new halloysite-rich clay deposits. Nevertheless, most previous research demonstrated results after small-scale lab experiments, something that should be changed in future investigations. Extended characterization of halloysite minerals in clay raw materials has to be provided in new investigations, since its characteristics and amounts strongly affected the processing and new products, thus leading to more safe conclusions as well as to up-scaling experiments too. Furthermore, synergistic halloysite-rich clays with secondary industrial materials and/or halloysite mining wastes should be investigated further in the future, as low-cost raw materials for the production of traditional ceramics under the global circular–green economy frame. Scientific interest on halloysite-based advanced ceramics was present (SiAlONS), although this does not remain at the forefront at this time, mainly due to high cost of treatments on a large scale, which could change in the future, especially if new technology is developed. Investigations on new different ceramic composites with low-cost HNTS and specific characteristics are in the spotlight currently and it seems that the scientific interest will rather increase the coming years. For this reason, future studies should include at least basic economic analyses in order to further increase the interest of the involved industries for future investment on these products.

## Figures and Tables

**Table 1:** Clay materials with halloysite for their potential use in traditional ceramics.

Origin of Halloysite	Starting Raw Materials	Basic Mineral Composition of Clay Raw Material	Characteristics of Halloysite (Type, Morphology, Particle Size, Amount in Raw Material)	Synthesis/Processing	Potential Use/ Ceramic Product	Ref.
Central Italy	Halloysite-rich clay	Halloysite, siliceous amorphous, feldspars, limonite, biotite, chabazite	Halloysite 10 Å, 48–70 wt%,	Sintering/crushing and grounding of samples. Pressing at 300 N/mm^2^. Slow firing (2 °C/min), at 1200 °C and remaining for 10 h, slow cooling inside the industrial kiln in 10 h. Rapid firing at 1085 or 1120 °C in 41 min and 1195 °C in 60 min, rapid cooling inside the kiln.	Mullite-based ceramic	Gualtieri and Bertolani (1992)
Troshkovsk (Russia)	Kaolinite–halloysite clay	Halloysite, alumina kaolinite, montmorillonite	10–40 wt%	Not provided	Refractory products	Shelest, (1992)
Not provided	Kaolinite-rich clay or halloysite-rich clay, magnesium hydroxide	Halloysite, accessories	Needle like halloysite, 0.5–1 μm in size	Sintering/mixtures of (i) kaolinite, synthesized magnesium hydroxide and (ii) halloysite, synthesized magnesium hydroxide sintered at 1350 °C at a rate of 100 °C/min in a reducing atmosphere	Cordierite Ceramics	Sumi et al. (1998)
Cameroon	Kaolinite–halloysite clay	Kaolinite, halloysite, illite, quartz, goethite, feldspar	Not provided	Not provided	Burnt bricks, ceramics, earthenware	Elueze et al. (2004)
Algeria	Halloysite-rich clay	Halloysite, accessories	Halloysite 10 and 7 Å	Spark plasma sintering/sintering at 1300 °C with a rate of 100 °C/min under pressure of 40 MPa in a reducing atmosphere	Nano-mullite-based ceramic	Imai et al. (2006)
New Zealand	Kaolinite-rich clay or halloysite-rich clay with or without alumina	Not provided	Not provided	Sintering/prepared starting mixtures pressed at 113 MPa by uniaxial pressing and at 120 MPa by cold isostating pressing. Heating at a maximum temperature of 1500 °C with a heating and cooling rate of 10 °C/min.	Mullite-based ceramic	Tezuka et al. (2006)
Indonesia	Halloysite-rich clay	Halloysite, feldspar, montmorillonite, mica, quartz	Not provided	Sintering/Halloysite-rich clay fired at 1100 and 1200 °C	Several traditional ceramic products and tiles	Septawendar et al. (2007)
Cameroon	Kaolinite–halloysite clay	Kaolinite, halloysite, quartz, feldspars, illite, carbonates, phosphates, iron hydroxides, anatase	Tubular halooysite 10 and 7 Å	Not provided	Conventional ceramics, earthen bricks	Pialy et al. (2008)
Patagonia	Halloysite-rich clay	Kaolinite–halloysite clay /kaolinite, halloysite, smectite, illite/smectite, quartz, feldspars	Tubular halloysite,	Not provided	Sanitary ware, porcelain, stoneware	Dondi et al. (2008)
Bulgaria	Halloysite-rich clay	Halloysite, sanidine	Not provided	Not provided	Tiles, bricks	Djambazov et al. (2009)
New Zealand	Kaolinite-rich clay or halloysite-rich clay, alumina, fluoride additives, oxide additives.	Halloysite, cristobalite, quartz	Not provided	Sintering/preparation mixtures of 50 wt% halloysite (or kaolinite), 50 wt% alumina, 1.5 or 3.0 wt% TiO_2_ additive or 2.5–5 wt% Fe_2_O_3_ additive. Pressing at 110–113 MPa by uniaxial pressing and at 120–200 MPa by cold isostating pressing. Heating at 1300–1600 °C with a heating rate of 1.5 °C/min.	Alumina/Clay ceramics	Tezuka et al. (2009)
New Zealand	Halloysite-rich clay, lithium hydroxide or lithium silicate	Halloysite, cristobalite, quartz	Not provided	Conventional method of geopolymer, solid state, thermal reactions, sintering/dehydroxylation of halloysite at 570 °C. Synthesis of lithium aluminosilicate precursors compounds: (i) after dehydroxylated halloysite with lithium hydroxide or lithium silicate at alkaline conditions or (ii) by solid state reaction of dehydroxylated halloysite with lithium hydroxide at 570 °C for 4 h or (iii) firing of dehydroxylated halloysite with lithium hydroxide, fine silica fune powder at 600 °C. Sintering of precursors up to 1275 °C.	Lithium aluminosilicate ceramics	Conor and MacKenzie (2010)
E. Algeria	Halloysite-rich clay, kaolinite-rich clay, chamotte	Halloysite, kaolinite, quartz, calcite, goetite, plagioclase	Not provided	Sintering/prepared mixtures of 30 wt% halloysite clay, 35 wt% kaolinite clay, 30 wt% chamotte. Pressing under 35 MPa and firing at 1350 °C for 33 h.	Refractory	Amrane et al. (2011)
N. Thailand	Kaolinite-rich clays, Kaolinite–halloysite clay, halloysite-rich clay, ball clay	Kaolinite, halloysite, illite, microcline, quartz, anatase, gibbsite	Hollow microtubules and plates of halloysite 7 Å, with dimensions 0.08–0.2 μm diameter and 0.50–4.5 μm length, 70 wt%	Sintering/prepared dry powders pressed under 5000 kg and firing from 880 to 1300 °C.	Ware ceramics, porous ceramics, inorganic polymer	Bordeepong et al. (2011), Bordeepong et al. (2012)
N. Tunisia	Kaolinite–halloysite clay, Clays with quartz-feldspar and low philosilicates	Halloysite, kaolinite	Halloysite7 Å < 2 μm, 59–61 wt%	Sintering/piles: prepared mixtures pressed at 250 bar and sintered at 800–950° (10 °C/min for 30 min). Bricks: prepared mixtures sintered at 950–1100° (15 °C/min for 30 min).	Bricks, tiles	Moussi et al. (2011)
Algeria (Guelma and Constantine region)	Halloysite-rich clay, calcite with or without methocel, amijel	Halloysite, accessories	Halloysite nano rodes, <1.21 μm in particle size, type, 70–90 wt%	Sintering/(i) mixing of halloysite-rich clay, 10–28 wt% calcite, compacting at 75 MPa, small sized discs configuration, sintering at 1300 °C for 1 h. (ii) Mixing of halloysite-rich clay, 15 wt% calcite, 4 wt% methocel, 3 wt% amijel, extrusion or roll pressing, tubular or flat configuration, sintering at 1100–1250 °C for 1 h.	Porous mullite-based ceramics	Harabi et al. (2014)
Commercial	(i) Kaolinite-rich clay, alumina, starches (potato or corn), (ii) commercial aluminosilicate powder of, starches (potato or corn)	Kaolinite, halloysite, talc, quartz, accessories	Tubular halloysite 10 and 7 Å	Consolidation, sintering/preparation of water-based ceramic–starch suspension by mixing of the aluminosilicate raw materias with starches and water (in a ratio of 60 solid/40 water). Consequent homogenizing for 4 h, deggasion in vacuum for 20 min and consolidation at 80 °C for 4 h. Heating at 1 °C/min up to 650 °C, 3 °C/min up to 1330 °C and cooling at 5 °C/min.	Ceramic with thermal insulating properties/porous cordierite-based ceramics	Gass et al. (2015)
W. Tunisia	Halloysite-rich clay	Halloysite, quartz, calcite, dolomite, smectite, iron and zinc oxides	Halloysite 10 Å, tubular or fibrous and seldom platelets, <2 μm particle size, 25–30 wt%	Sintering/prepared mixture of 95 wt% halloysite clay, 5 wt% dolomite. Sintering up to 1050 °C with a heating rate of 5 °C/min	Tiles and refractory	Jemai et al. (2015)
East Algeria	Halloysite-rich clay	Halloysite, nacrite, gibbsite, chlorite, quartz kaolinite, hematite, todorokite, dickite, magnetite	Halloysite 10 Å, tubular halloysite and tubular rods 63–66 wt%	Not provided	Several ceramic applications	Senoussi et al. (2016)
Algeria	Halloysite rich clay, calcite	Halloysite, illite, calcite, quartz	Hollow nano-rods halloysite 7 Å, <1 μm in particle size	Sintering/calcination of CaCO_3_ at 900° C for 12 h. Prepared mixtures of 80 wt% halloysite-rich clay and 20 wt% CaO (produced after calcite calcination). Milling for 17 h, using a vibratory milling system and calcination at 800 °C for 1 h. Prepared discs after pressing under 75 MPa. Sintering at 800–1100 °C for 1 h.	Anorthite-based ceramics	Zaiou et al. (2016)
W.-S. Sinai, Egypt	Kaolinite–halloysite clay	Kaolinite, halloysite, dickite, quartz, smectite, illite, gypsum, dolomite, hematite	Not provided	Not provided	Ceramic products, Portland cement, refractory products, glaze manufacturing	El-Kammar et al. (2017).
E. Algeria	Halloysite-rich clay, boehmite, zirconia	Not provided	Halloysite 10 Å with platty or irregular shape, 10–50 μm particle size	Sintering /preparation of mixtures with halloysite-rich clay, boehmite and zirconia. Pressing under 75 MPa and firing at 1250–1650 °C for 2 h.	Mullite–zirconia ceramics	Raghdi et al. (2017)
Algeria	Kaolinite–halloysite clay, ΒaCO_3_	Kaolinite, halloysite, traces of organic matter	14 wt%	Sintering/preparation of mixtures with clay and 0–60 wt% ΒaCO_3_. Pressing under 55 MPa. Sintering at 1100 and 1200 °C for 3 h, with a heating rate of 10 °C/min.	Barium aluminosilicate ceramics	Bouzidi et al. (2018)
Nador (NE Morocco)	Halloysite-rich clay	Halloysite, gibbsite, alunite, K-feldspar, smectite, illite	Tubular halloysite 7 Å, seldom agglomerated, 0.15 μm diameter, 600 μm length, ~70 wt% and	Sintering/Prepared specimens pressed under 100 MP and heated up to 1100 °C.	Refractory products	Haddar et al. (2018)
New Zealand	Halloysite-rich clay, kaoline-rich clay, feldspar flux, quartz sand	Halloysite kaolinite, minor quartz.	Tabular 7 Å, 0.3 μm in particle size, 88 wt%	Sintering /preparation of mixtures with 53 wt% halloysite-rich clay or kaoline-rich clay, 33 wt% feldspar and 14 wt% quartz sand. Sintering in an electric kiln at 1270, 1300, 1320 and 1340 °C with a fast (5°/min for 90 min) and a slow (2°/min for 180 min) heating rate.	Porcelain	Sanz et al. (2018)
NE Morocco	Halloysite-rich clay, diatomite, marl, silica sand, recycled alumina	Halloysite, amorphous, K-feldspar, amorpous phase, traces of tridymite, alunite, fluorite, halite, illite, smectite, diopside, gibbsite	Halloysite 7 Å, 68 wt%	Sintering/Preparation of mixtures with raw materials pressed under 30 MPa and firing up to 1300 and 1500 °C in an electric furnace with a heating and cooling rate of 3 and 5 °C/min	Refractory	Haddar et al. (2019)
Korea, China, Vietnam	Kaolinite–halloysite clays	Kaolinite, muscovite feldspar, halloysite, rutile, gibbsite, quartz	Needle-like halloysite, <1 μm in diameter diameter, ~5 wt%	Sintering/specimens heated at 1200 and 1250 °C	Porcelain	Kim and Hwang (2019)
China	Halloysite-rich clay, calcined talc, α-Al_2_O_3_ powder, MgO	Halloysite, accessories	Tubular halloysite 10 Å	Sintering/prepared mixtures of raw materials were pressed under 10 MPa and were fired at 1200–1400 °C for 2 h in a silicon–molybdenum furnace.	High temperature thermal storage applications/cordierite ceramics	Lao et al. (2019)
Algeria	Halloysite-rich clay, kaoline-rich clay, ZrO_2_	Halloysite, minor quartz and orthoclase	Nano-rod halloysite ~20 wt%	Sintering/preparation of mixtures with 5 and 8% ZrO_2_ additive. Sintering up to 1250 °C.	Porcelain	Serragdj et al. (2019)
E. Algeria	Halloysite-rich clay and/or kaolinite–rich clay, fireclay, talc	Halloysite, minor albite, orthoclase, calcitehematite	90 wt%	Sintering/prepared mixtures of raw materials were pressed under 250 bars and were fired up to 1330 °C for 43 h in an intermittent oven	Mullite–cordierite Refractory	Cheraitia et al. (2021)

**Table 2:** Use of halloysite in advanced ceramic products.

Origin of Halloysite	Starting Raw Materials	Synthesis	Processing	Ceramic Product	Ref.
Not provided	Kaolinite-rich clay, halloysite-rich lay, carbon	Carbothermal method and nitridation method	Firing of starting mixtures at 1800 °C for short periods	SiAlON	Lee et al. (1976)
China	Halloysite-rich clay or kaolinite-rich clay, carbon	Carbothermal reduction and nitridation method	Starting mixtures milled in hexane for 17 h and fired at 1400 °C for times 10 min to 24 h	β’-SiALON	MacKenzie et al. (1994)
New Zealand	Halloysite-rich clay, carbon	Carbothermal reduction and nitridation method	Firing of starting mixtures at 1400 °C for 1–480 min	β-SiAlON	Neal et al. (1994)
China	Halloysite-rich clay, carbon, silica or elemental Si ± Y_2_O_3_	Carbothermal reduction and nitridation method	Starting mixtures milled for 24 h in ethanol and fired at 1000–1400 °C for 2 h	low-z β’-SiAlON	MacKenzie et al. (1997)
New Zealand	Halloysite-rich clay, carbon, silica or elemental Si, Y_2_O_3_	Carbothermal reduction and nitridation method	Starting mixtures milled in ethanol for 4 h and fired at 1475 °C for 8 h, (a-SiAlON powders)/α-SiAlON powders were pelletized and hot pressed at 1800 °C for 2 h (dense α-SiAlON ceramics)	a-SiAlON	Ekstrom et al. (1998)
China	Halloysite-rich clay, alumina, elemental Si, metal oxides additives	Silicothermal method and nitridation method	Starting mixtures milled for 20 h in hexane using Si_3_N_4_ media and fired at 1200–1500 °C, with the remaining 4 h at the maximum temperature	X-SiAlON	Sheppard and MacKenzie (1999)
New Zealand	Halloysite-rich clay, talc, carbon black± 3 wt%α-Si_3_N_4_	Carbothermal reduction and nitridation method	Starting mixture milled in ethanol for 24 h and fired at 1450–1500 °C in nitrogen gas for 2–16 h	Mg-αSiAlON	Qiu et al. (2002)
New Zealand	Halloysite-rich clay, fine carbon	Carbothermal reduction and nitridation method	Starting mixture ground for various periods and heated at 1200–1400 °C for 12 h	β-SiAlON	MacKenzie et al. (2006)
Not provided	Halloysite-rich clay, Si, AlN	Silicothermal reduction and nitridation method	Sintering of starting prepared mixtures at maximum temperature of 1250–1500 °C for 3 h	β-SiAlON	Yin and Jones (2017)

**Table 3:** Use of halloysite in “ceramic composites”.

Origin of Halloysite	Synthesis	Processing	Product	Application	Ref.
New Zealand	Electroless deposition/heat tretment	Activated tubular halloysite ceramic substrate by Pd particles and electroless deposition of Ni nanoparticles on halloysite and heat treatment at 400 °C	Metallized-ceramic material	Used as magnetic material	Fu et al. (2004)
New Zealand	Electroless deposition/heat treatment	Activated tubular halloysite ceramic by Pd particles and electroless deposition of Ni nanoparticles and wires on s halloysite template and heat treatment at 400 °C	Metallized-ceramic material	Used as magnetic material	Fu and Zhang (2005)
China, New Zealand	Pelletization	Halloysite with ethylcellulose, ethanol and sucrose were mixed for 12 h at room temperature and pelletized by extrusion (under extruder rotation 30 rpm) and spheronization (under sheronizer speed and time 1500 rpm and 10 min, respectively)	Porous aluminosilicate ceramic pellets	Used as a drug delivery vehicle	Byrne and Deasy (2005)
New Zealand	Pelletization	Halloysite, microcrystalline cellulose and a fentanyl base were mixed for 10 min. Powder was mixed with water(forming paste). Paste extruded as spaghetti-like threads which were spheronized to pellets with 1.5 mm diameter and, consequently, they were dried for 24 h.	Ceramic drug delivery vehicle	Used as a drug delivery vehicle for release of potent opioids	Forsgren et al. (2010)
USA	Mixing and centrifugation/filtering	Loading of halloysite in a solution of inhibitors by consequent vacuation in 3 cycles. Separation of loaded tubes from the solution by centrifugation or filtering, drying at 60–70 °C and grinding. Mixing of loaded halloysite with a solution of polymers.	Ceramic–polymer composite	Used as anticorrosion coating of alloys	Fakhrullin et al. (2014)
New York	Deposition and vacuum evaporation	Halloysite nanotubes were deposited on aluminum foil. A thin metal film of 15 nm was coated on an aluminum-halloysite substrate in a vacuum evaporator	Al-ceramic HNTS substrate	Substrate for surface enhanced raman spectroscopy	Vinokurov et al. (2015)
New Zealand	Electrospinning and sintering	Preparation of halloysite–polyvinyl pyrrolidone nanofiber mats by electrospinning (at 120–240 mm distance of the spinning and collecting electrode, 0–80 kV voltages, ~40% humidity). Sintering of the mats at 1100 °C in N_2_ and 1200–1400 °C in air	Ceramic nanofiber mat	Support for zeolite membranes	Chen et al. (2016).
USA	Sintering	Mixing of halloysite with carbon fibers and lubricating agent for 15 min. Pressing samples with 30 mm diameter under 100 MPa at 20 °C. Sintering at 1500 °C and air atmosphere.	Porous ceramic preform	Used in metal matrix synthesis for novel applications	Kujawa et al. (2016)
Commercial	Sintering, gas pressure infiltration	(I) Synthesis of porous mullite performed: mixture prepared of halloysite powder and 30 wt% carbon fibers. Pressing at 100 MPa. Sintering at 1300 °C. (II) Infiltration of aluminium alloy into the porous perform matrix under nitrogen pressure of 3 MPa in 3 s.	Metalized-porous ceramic composite	Used for advanced applications, especially under high temperatures	Pawlyta et al. (2016)
Commercial	Modification, dispersion, electrospinning	Modification of halloysite nanotubes (HNT-NH2, HNT-COOH) after reactions of HNT with APTES and maleic anhydrite. Preparation of drug-loaded halloysite in appropriate solutions and stirring at 37° for 24 h. Dispersion of neat HNT, HNT-NH_2_, HNT-COOH into an electrospun substrate of PCL (polycaprolactone) under 25 °C and 13–14 kV. In vitro release of drug load.	Polymer–ceramic nanocomposites	Used as a drug release carrier	Ghaderi-Ghahfarrokhi et al.(2017)
New York	Schiff base binding	Production of halloysite nanotubes by rolling aluminosilicate sheets. Mixing of halloysite tubes with furfuraldehyde and ultrasonic dispersion for 30 min. Mixing of furfuraldehyde loaded halloysite with hydrazine hydrate and stirring for 30 min at 70 °C forming a Schiff base.	Metal–ceramic core–shell composite	Industrial applications of metal nanocatalyst or as metal adsorbent from solutions	Vinokurov et al. (2017)
China	Sintering	Preparation of three different mixtures of MVQ/HNT with different borates as additives: mixing time 20 min, pressing under 15 MPa at 170 °C for 15 min. Heating at 1000 °C for 30 min.	Ceramizable silicone rubber (MVQ)/halloysite (HNT) composite	Special application as fire-resistant wires and cables	Guo et al. (2018)
China	Microwave sintering	Mix of low-grade pyrite cinder (LPC) with coal powder (in ratio 10/1) and 5% calcium fluoride. Preparation of discs after pressing and sintering in ab electric furnace up to 1450 °C (5 °C/min and slow cooling in a room)	Microwave heating ceramics (MHCs)	Used as magnetic ceramic and aggregate for asphaltic mixtures	Zhang et al. (2018)
Commercial	Plasma electrolytic oxidation (PEO)	PEO coating of AM50 alloy was prepared in an alkaline electrolyte (at 20 °C and frequencies ranging from 100 to 5000 Hz) and consisting of HNTS, Na_2_SiO_3_, KOH, NaF	Incorporated halloysite into forsterite ceramic coating	Used as coating	Mingo et al. (2019)
Commercial	Electrophoretic deposition (EPD)	Mixture of chitosan (CS), bioglass (BG), hydroxyapatite (HA) and halloysite nanotubes (HNT) powders added to ethanol–water solvent and stirred for 24 h at room temperature. Deposition on the titanium substrate at voltages of 10–50 kV and times of 5–25 min	Chitosan-based nanocomposite	Used as coating of metallic substrate	Molaei et al. (2019)
USA	Metal deposition/casting method	2 wt% metal of Pd, Pt derived from chemical solutions with H_2_PtCl6 and salt PdCl_2_, laid down in the halloysite tubes. 4 wt% of the hybrid Pd, Pt /HNT mixed with sulfopolymer solution in isopropanol or dimethylformamide with a magnetic stirrer for 30 min.	Metal–ceramic shell halloysite syntheses in hybrid membranes	Used as a separating membrane in fuell cells or in an electromembrane	Petrova et al. (2019)
Commercial	Sulphuric acid treatment, sol–gel method and heating	Etching of Al_2_O_3_ in HNTS by sulphuric acid at 70 °C for 20 h producing SiO_2_ (SiNP). Sol–gel synthesis of clay-based Na_2_ZnSiO_4_ (Clay NZS) using a mixture of SiNP, sodium acetate, zinc acetate dehydrate. Drying for 24 h, calcining at 350 °C for 6 h, pressing at 2 tons and sintering at 700, 750 and 800 °C	Ceramic clay-based Na_2_ZnSiO_4_ compound	Used as ceramic electrolyte	Johari et al. (2020)
Commercial	Plasma electrolytic oxidation (PEO)	HNTS loaded with corrosion inhibitors (vanadate, molybdate, 8-hydroxyquinoline) after mixing with a aqueous solution under a vacuum. PEO coating of AZ31 magnesium alloy was prepared in an alkaline electrolyte (at 20 °C and frequencies up to 5000 Hz) and consisting of HNTS, Na_2_SiO_3_, KOH, NaF. PEO-coated and immersed in an aqueous solution with 20 gr/L loaded HNTS for 10 min, at 22 °C, pH values under 6.5–7.5.	Smart ceramic coating	Used as coating	Mingo et al. (2020)

## Data Availability

The data presented in this review study are available on request from the corresponding author.
